# Nitrofurantoin: what is the evidence for current UK guidance?

**DOI:** 10.1093/jac/dkad287

**Published:** 2023-09-20

**Authors:** Eleanor Kashouris, Amelia Joseph, Tom Lewis

**Affiliations:** Department of Sociology and Criminology, University of Sussex, Falmer, Freeman Building, Brighton, BN1 9RH, UK; Department of Clinical Microbiology, Nottingham University Hospitals NHS Trust, Nottingham, NG7 2UH, UK; Department of Microbiology, Royal Devon University Hospital, Barnstaple, EX31 4JB, UK

## Abstract

Three days of nitrofurantoin at 100 mg twice daily is recommended as first-line treatment for uncomplicated urinary tract infection (UTI) in evidence-based guidelines across the UK. A review of international guidelines shows that the evidence base has been interpreted in very different ways. UK guidelines are unusual in promoting short (3 day) courses, and we find little direct evidence to support this. Although ‘short’ courses of antibiotics for other agents may provide optimum balance between providing effective treatment whilst reducing selective pressure driving resistance amongst colonizing microbial flora, it remains unclear that course lengths can be extrapolated to nitrofurantoin. Three days of nitrofurantoin may indeed be a useful intervention in a large group of patients. However, without supporting evidence and because clinical response should be expected to vary widely, it is unclear that establishing and promoting an antibiotic duration for UTI is the best approach to optimizing prescribing in this important area.

## Introduction

Urinary tract infection (UTI) is one of the most commonly seen bacterial infections in general practice,^1^ and the proportion of prescriptions for UTI that are identified as appropriate is higher than for other bacterial infections.^2^ Meanwhile, UTI is the largest cause of community-onset bacteraemia,^3^ and effective management of community-acquired UTIs in adults may be important in reducing rates of sepsis.^4^ Uncomplicated UTI has therefore become a key topic area for antimicrobial stewardship interventions. The consequences of suboptimal management in terms of persistence or recrudescence of infection also remain unclear.^5,6^ Evidence-based guidelines attempt to optimize prescribing for UTI by standardizing antibiotic therapy to a set duration.

Oral options for treating uncomplicated UTI are limited by increasing antibiotic resistance to trimethoprim and safety concerns surrounding fluoroquinolones. In the UK, this has led to an increased use of nitrofurantoin.^7^ Nitrofurantoin provides good empirical cover of uropathogens, reaches high concentrations in the bladder^8,9^ and has a low impact on endogenous resident microflora.^10^ Moreover, a greater proportion of trimethoprim prescribing to nitrofurantoin prescribing at general practice level in adult women was significantly associated with a higher incidence of trimethoprim-resistant *Escherichia coli* bacteraemia in bacteraemias reported from January 2012 to December 2014 to the national mandatory surveillance programme.^11^ In 2016/17, a reduction in the trimethoprim to nitrofurantoin prescribing ratio for uncomplicated UTI was incentivized through the NHS England Quality Premium.^12^ This incentivization contributed to increasing the use of nitrofurantoin for 3 days, in line with UK national guidelines.^4,13^

In this article, we compare the recommendations made on the use of nitrofurantoin in guidelines internationally. In the context of widespread variation in clinical response and tensions between under-treating and over-treating, we analyse how the evidence base has been interpreted in guidance on how best to manage uncomplicated UTI.

## Methodology

We conducted a review of guidelines for the use of nitrofurantoin internationally in the treatment of uncomplicated lower UTI. The aim of the review was to capture the level of variation globally surrounding recommendations on nitrofurantoin.

Mixed methods were utilized in order to identify guidance, searching for the latest version of guidelines on lower UTI published between January 2010 and January 2023. This was because no one search method consistently yielded relevant and specific results. In PubMed and Google Scholar, a combination of different search terms using the terms ‘urinary tract infection’ or ‘UTI’ or ‘cystitis’ and ‘guidance’ or ‘guideline’ or ‘recommendation’ was used. Results pages, filtered by relevance, were searched until results consistently became no longer relevant. Repositories of national clinical guidelines were searched using the terms ‘urinary tract infection’ or ‘UTI’ or ‘cystitis’, specifically the Guidelines International Network and Guideline Central. The official webpages of major uro-gynaecological, microbiological, infectious diseases and general practice organizations were searched. Reference lists of previous research and guidelines were also searched for citations of guidelines.

Uncomplicated UTI was defined according to the European Association of Urology classification, which is limited to ‘non-pregnant women with no known relevant anatomical and functional abnormalities within the urinary tract or comorbidities.’^14^ Lower UTI was defined as an infection of the urinary bladder. Guidance related to recurrent UTI, upper UTI and complicated UTI was excluded. Guidance published in languages other than English were translated by E.K. No guidance, once identified, was unable to be translated. The search was not restricted by country but it is possible and indeed probable that guidelines, especially those written in languages other than English, were not captured by this English-language search. However, the aim of comparing recommendations on nitrofurantoin globally to ascertain the level of variation in guidance internationally was achieved.

Identifying outlying recommendations on nitrofurantoin course length in some guidance, we then reviewed and analysed the evidence cited in support of these outlying recommendations. Firstly, this involved identifying which studies included in the systematic reviews cited had arms using nitrofurantoin as an intervention. Secondly, we evaluated these arms individually for their relevance and applicability to the recommendation made in terms of antibiotic agent, dosage and duration. No comparative methodology to compare between studies for quality of evidence was necessary given the nature of the findings.

Finally, we discuss evidence pertaining to outlying nitrofurantoin interventions that was not cited in the guidance recommending these interventions. This was in order to discuss the recommendation itself, based on available evidence, as well as to evaluate the evidence upon which recommendations were based. Evidence was previously known to the authors and mixed methods searches yielded no further relevant results.

## Findings

### Comparison of recommendations on use of nitrofurantoin across international guidelines

Twenty-seven guidelines from major organizations internationally were identified. There is widespread variation in recommendations on nitrofurantoin use globally (Table 1). In particular, UK guidelines from NICE and the Scottish Intercollegiate Guidelines Network (SIGN), Norwegian guidance from the Directorate for Health, and Finnish guidance are outliers in recommending shorter courses of nitrofurantoin. Both NICE^15^ and SIGN^16^ recommend a 3 day course of nitrofurantoin 100 mg modified-released (MR) twice daily or 50 mg four times a day as first-line treatment for women presenting with symptoms of uncomplicated UTI who are assessed to be in need of antibiotics. The Norwegian Directorate for Health recommends a 3 day course of nitrofurantoin 50 mg three times daily.^36^ The Finnish Medical Association recommends a 3 day course of 75 mg twice daily.^37^ The German College of General Practitioners and Family Physicians guideline also recommends considering a 3 to 5 day course of 100 mg twice daily.^27^ The Belgian EBM PracticeNet guideline^31^ recommends a 3 to 5 day course of nitrofurantoin but the dose is higher, with 100 mg recommended three times daily. Meanwhile, most other guidance internationally recommends longer courses of nitrofurantoin. A major international guideline from the IDSA and ESCMID jointly recommends a 5 day course of 100 mg twice daily,^18^ and the European Association of Urology (EAU)^14^ recommends a 5 day course of 50–100 mg four times daily, or 100 mg (MR) twice daily.

**Table 1.** Comparing recommendations on uses of nitrofurantoin between different guidelines

Guideline organization	Last updated	Country or region	Recommended use of nitrofurantoin	Duration	Dose
National Institute for Health and Care Excellence (NICE)^15^	2018	England, Wales and N. Ireland	First line	3 days	100 mg twice daily or 50 mg four times daily
Scottish Intercollegiate Guidelines Network (SIGN)^16^	September 2020	Scotland	First line	3 days	100 mg twice daily or 50 mg four times daily
Nederlands Huisartsen Genootschap [Dutch General Practitioners Association]^17^	April 2020	Netherlands	First line	5 days	100 mg twice daily or 50 mg four times daily
IDSA and ESCMID^18^	2010 (under review)	USA and Europe	First line	5 days	100 mg twice daily
Asociación Española de Urología [Urology Association of Spain]^19^	April 2017	Spain	Second line	5 days	100 mg twice daily
La Sociedad Española de Enfermedades Infecciosas y Micobiologogía Clínica [Spanish Society of Clinical Microbiology and Infectious Diseases]^20^	May 2017	Spain	First line	5–7 days	Not specified
European Association of Urology (EAU)^14^	2021	Europe	First line	5 days	50–100 mg four times a day or 100 mg twice a day
Société de Pathologie Infectieuse de Langue Française (SPLIF) [French Infectious Disease Society]^21^	May 2018	France	Nitrofurantoin not recommended due to risk of toxicity		
Swiss Society of Gynaecology and Obstetrics (SSGO)^22^	2020	Switzerland	First line	5 days	100 mg twice daily
Société Suisse d’Infectiologie [Swiss Society of Infectious Diseases]^23^	2014	Switzerland	First line	5 days	100 mg twice daily
Läkemedelsverket [Swedish Medical Products Agency]^24^	4 March 2019	Sweden	First line	5 days	50 mg three times daily
Svenska Infektionsläkarföreningen [Swedish Association of Infectious Diseases]^25^	2020	Sweden	First line	5 days	50 mg three times daily
Korean Centre for Disease Control and Prevention^26^	2018	Korea	First line	More than 5 days	100 mg twice daily
Deutsche Gesellschaftfür Allgemeinmedizin und Familienmedizin (DEGAM) [German College of General Practitioners and Family Physicians]^27^	August 2018	Germany	First line	3 to 5 days	100 mg twice daily
Deutsche Gesellschaft für Urologie [German Society for Urology]^28^	April 2017 (expired, under review)	Germany	First line	7 days	50 mg four times daily
UAA (Urological Association of Asia) and AAU (Asian Association of UTI and STI)^29^	2017	Asia	First line	5 to 7 days	100 mg twice daily
Società Italiana di Urologia [Urological Society of Italy]^30^	2015	Italy	First line	5 days	50–100 mg four times daily
EBM PracticeNet Werkgroep ontwikkeling richtlijnen eerste lijn [EBM PraticeNet Working Group for the Development of Primary Care Guidelines]^31^	October 2016	Belgium	First line	3–5 days	100 mg 3 times daily
Associação Médica Brasileira e Agência Nacional de Saúde Suplementar [Medical Association of Brazil and National Agency of Supplementary Heath]^32^	January 2011	Brazil	First line	7 days	100 mg four times daily
Arbeitsgemeinschaft der Wissenschaftlichen Medizinischen Fachgesellschaften (AWMF) [Association of Scientific Medical Societies]^33^	2017	Germany	First line	5 days OR 7 days	100 mg twice daily 50 mg four times daily
Indian Council of Medical Research^34^	2019	India	First line	5 days	100 mg twice daily
Ministry of Health of the Republic of Serbia^35^	2022	Serbia	First line	5 days	100 mg twice daily
Helsedirektoratet [Norwegian Directorate of Health]^36^	September 2022	Norway	First line	3 days	50 mg three times daily
Käypä hoito [Finnish Medical Association]^37^	2021	Finland	First line	3 days	75 mg twice daily
Ministry of Health of Russia^38^	2016	Russia	First line	5 days	100 mg three times daily
Qatari Ministry of Public Health^39^	June 2020	Qatar	First line	5 days	100 mg four times daily

### Evidence cited for outlying guidance: NICE, SIGN, Norwegian and Finnish guidance

A Cochrane Library systematic review of evidence comparing the efficacy of 3 day courses with 5 day courses of antibiotics^40^ is presented by NICE, SIGN and the Norwegian Directorate for Health as evidence for ‘short’ courses of 3 days of nitrofurantoin for treating acute UTI in non-pregnant women. The equal efficacy of 3 day and 5 day courses was a finding independent of antibiotic class used. However, the review did not include any trials that had nitrofurantoin courses of less than 7 days as a study arm. As noted by the authors, the drug in the 3 day group was a quinolone in nearly all of the studies, with no direct evidence for the use of 3 days of nitrofurantoin 100 mg twice daily, the current recommended therapy in the UK for acute uncomplicated UTI in women.

The Norwegian Directorate for Health presents no trial evidence on the choice of antibiotic, citing *in vitro* resistance rates. NICE cites a meta-analysis of randomized controlled trials,^41^ and two Cochrane systematic reviews,^42,43^ as evidence supporting nitrofurantoin as first-line antibiotic choice for non-pregnant women. SIGN cites one of these Cochrane systematic reviews.^42^ The meta-analysis compared fosfomycin with 7 day courses of nitrofurantoin and found no significant difference.^41^ One of the Cochrane reviews compared trials involving quinolones only.^43^ The other Cochrane review^42^ included one small clinical trial,^44^ where 3 days of nitrofurantoin was compared with 3 days of trimethoprim/sulfamethoxazole 160/800 mg twice daily, 3 days of cefadroxil 500 mg twice daily, and amoxicillin 500 mg twice daily. Not only was nitrofurantoin found to be inferior in terms of clinical cure to trimethoprim/sulfamethoxazole (61% versus 82%; *P *= 0.05), but the dosage of nitrofurantoin used in the study was 100 mg four times daily, double that of current UK recommendations.

Therefore, the current UK recommendation for a 3 day course of 100 mg nitrofurantoin MR twice daily is based on evidence of the equal effectiveness of 3 day quinolones and 5 day courses of other antibiotics,^40^ and of the equal effectiveness of 7 days of nitrofurantoin to fosfomycin^41^ and β-lactams and trimethoprim/sulfamethoxazole.^42^ No direct evidence on the clinical efficacy of 3 days of 100 mg nitrofurantoin MR twice daily (or 50 mg immediate release four times daily) is presented by NICE, SIGN or the Norwegian Directorate for Health’s evidence review. There is some evidence on 3 days of 100 mg nitrofurantoin four times daily, which demonstrated it to be inferior to 3 days of amoxicillin, cefadroxil and trimethoprim/sulfamethoxazole.^42,44^

The limitations of the evidence base are not recognized in the guidance of NICE or SIGN. In contrast, previous SIGN guidance on UTIs from 2012 noted that the recommendation of 3 days of nitrofurantoin differed from IDSA recommendations and that ‘there is no direct evidence comparing three days nitrofurantoin with seven days nitrofurantoin.’^45^

The Finnish guideline does not cite systematic reviews but recommends a 3 day course of nitrofurantoin on the basis that ‘the microbiological and clinical responses achieved with nitrofurantoin do not differ from each other in different studies where treatment times have been either 3, 5 or 7 days. A direct comparison of nitrofurantoin treatments of different lengths has not been made.’^46^ The data on 3 day courses come from the small US clinical trial analysed above^44^ using 3 days of nitrofurantoin at a dosage of 100 mg four times daily, and from a 2002 study from Belgium^47^ also using 3 days of nitrofurantoin at a dosage of 100 mg four times daily. The study found that a 3 day course of nitrofurantoin was significantly more effective than placebo for uncomplicated UTI but noted that it was ‘surprising to have so few patients in the treated group with completely resolved symptoms after three days’ with 37% symptomatic cure, and that ‘patients should be informed about the frequent persistence of some symptoms after three days of nitrofurantoin treatment, despite the apparently successful bacteriological eradication.’ Therefore, there is no direct evidence for the Finnish recommendation of 75 mg nitrofurantoin twice daily for 3 days nor is there evidence directly comparing 3 day courses of nitrofurantoin with longer courses.

### Additional evidence on 3 day courses of nitrofurantoin

There is some, albeit very little, evidence available on 3 day courses of nitrofurantoin in addition to that cited in guidance that recommends this intervention. The Belgian trial^47^ cited in the Finnish guidance is not cited by NICE, SIGN or the Norwegian guideline. As in the US trial,^44^ the dosage in this trial, which showed 37% symptomatic cure after 3 days of treatment with nitrofurantoin, is double that of current UK guidance, at 100 mg four times daily.

A systematic review and meta-analysis in 2015^7^ (using data on 3 day courses of nitrofurantoin^44,47^) demonstrated equivalent clinical efficacy of nitrofurantoin to trimethoprim/sulfamethoxazole, ciprofloxacin and amoxicillin when used for 5 or 7 days (79%–92%), but diminished clinical efficacy when used for 3 days (61%–70%). This review was not prioritized by NICE, as the Cochrane review^42^ (which included one of the trials with a 3 day nitrofurantoin arm^44^ included here but not the other) was established to be of higher quality by the committee using NICE’s interim process guide.^48^ Neither systematic review provides evidence that a 3 day course of nitrofurantoin 100 mg twice daily is clinically equivalent to a 5 or 7 day course, or alternative antibiotics.

Evidence on 3 day courses of nitrofurantoin was published following high re-prescription rates in the Netherlands between 1992 and 1997.^49^ Treatment failure was defined as re-prescription of a common urinary antibiotic (trimethoprim, nitrofurantoin, norfloxacin, co-trimoxazole, amoxicillin or ofloxacin) within 31 days of finishing initial treatment. Results showed increased treatment failure of 18.9% after 3 days of nitrofurantoin, compared with 13.1% at 5 days and 12.5% at 7 days. This led the authors to conclude that 3 day courses of nitrofurantoin were less effective than 5 or 7 day courses.

## Discussion

**‘**Short’ courses of antimicrobial therapy are intended to be the shortest course length that maintains clinical efficacy. This optimizes the benefit of the antibiotic to the patient whilst minimizing side effects and reducing selective pressure driving antimicrobial resistance amongst microbial flora.^50,51^ The challenge in this approach is to identify the shortest course that will be clinically effective for the most people. However, both microbiological clearance and symptomatic resolution may be poor measures of clinical efficacy, even in patients who did have bacterial infection, as symptoms may be caused by residual inflammation.

We have been unable to identify any trial evidence on the clinical efficacy of the current recommended first-line therapy in the UK for uncomplicated UTI. NICE extrapolates that 3 days of nitrofurantoin is non-inferior to longer courses of nitrofurantoin because this is the case for other antibiotics. Antibiotics, however, vary in their wide range of different pharmacokinetics and modes of action. It is therefore not clear that course lengths can be extrapolated between drug classes without supporting clinical evidence.

The variation between recommendations internationally reflects both the uncertain foundations upon which evidence-based guidelines are written, and the limits of what can be safely concluded from evidence. A conclusion that there is more evidence for longer courses of nitrofurantoin than shorter, 3 day courses, ignores the paucity of evidence for either, and that the total absence of any evidence for current recommended practice means that comparisons cannot be made. Looking ahead, the National Institute for Health and Care Research (NIHR)-funded DurATIon-UTI trial, which started in August 2022 at the University of Oxford, UK, and is set to conclude in August 2025, will provide evidence on clinical efficacy of durations of 1, 2, 3, 4 and 5 day courses of nitrofurantoin, at currently recommended dosages.^52^

Evidence-based clinical guidelines that recommend antibiotic durations by indication are useful in primary care where ongoing assessment is limited compared with hospital settings. However, the concept of a course length in itself is limited. Evidence suggests that different patients respond differently to the same antibiotic.^40–43^ Genetic bacterial resistance and diagnostic uncertainty notwithstanding, this could be due to multiple host and pathogen factors such as individual immune response, microbiotic make-up^53^ and bacterial virulence.^54,55^ Moreover, response could well differ across different episodes of infection in the same patient. A study from 1984 compared single-dose (50 mg four times in 1 day) nitrofurantoin with nitrofurantoin 50 mg four times daily for 10 days.^56^ Single dose and 10 days were equally effective (95%–97% cure rate). It could be that in some patients with lower UTI, very short courses of even a single dose may be as effective as longer courses. In other patients though, 3 day courses are clearly ineffective. Treating for longer may resolve symptoms in a larger proportion of patients, although it may of course involve over-treating others, and data on longer courses of nitrofurantoin still demonstrate considerable rates of treatment failure.^57–60^ One possible explanation for this could be that whereas nitrofurantoin retains *in vitro* efficacy against a large proportion of urinary isolates derived from clinical settings, this may not correlate well with *in vivo* efficacy.

We join others^50,51,61^ in pointing out there may be ways of customizing duration of therapy to the patient’s response in primary care settings. Patients could be advised to contact the prescriber if symptoms resolve prior to finishing the course or else simply to stop treatment when they feel better. Another way of incorporating the expectation of individual variance in clinical efficacy into guideline care would involve a greater commitment to sharing uncertainty with clinicians and patients.^62^ Such efforts are underway in the WikiGuidelines initiative,^63^ which aims to ‘create guidelines with the humility of uncertainty.’ Pushing for greater compliance towards predefined course durations without the evidence to support it, especially without sharing the uncertainty of the evidence base, may risk a loss of confidence in recommended interventions. Certainly, there is a need for better safety-netting and guidance on the common clinical scenario of treatment failure in uncomplicated UTI.
